# Genetic Basis of Myxomatous Mitral Valve Disease in Cavalier King Charles Spaniel Dogs—A Review

**DOI:** 10.3390/vetsci12121144

**Published:** 2025-12-01

**Authors:** Maksymilian Lewicki, Sylwia Barbara Górczyńska-Kosiorz, Piotr Frydrychowski, Zuzanna Sidoruk, Agnieszka Noszczyk-Nowak

**Affiliations:** 1 Department of Internal Medicine and Clinic of Diseases of Horses, Dogs and Cats, Wrocław University of Environmental and Life Sciences, Grunwaldzki Sq. 47, 50-366 Wrocław, Poland; piotr.frydrychowski@upwr.edu.pl (P.F.); zuzanna.sidoruk@upwr.edu.pl (Z.S.); 2 Department of Internal Medicine, Diabetology and Nephrology, Faculty of Medical Sciences in Zabrze, Medical University of Silesia, 40-055 Katowice, Poland; skosiorz@sum.edu.pl

**Keywords:** MMVD, CKCS, NEBL, RAAS, SNP, gene, cardiology

## Abstract

Myxomatous mitral valve disease (MMVD) is the most prevalent cardiac disorder in small and toy breed dogs, with the Cavalier King Charles Spaniel (CKCS) showing exceptionally high predisposition and early onset of the disease. MMVD is characterized by progressive mitral valve degeneration, volume overload, and eventual development of congestive heart failure (CHF). Given the strong hereditary component in CKCS, considerable research has focused on elucidating the genetic basis of MMVD in this breed. This review article summarizes the current state of knowledge on the phenotypic features, inheritance, and candidate loci potentially responsible for early onset and severe course of the disease. The pathogenesis of the disease, its classification, and the effects of breeding programs aimed at reducing the occurrence of MMVD have been described. Key findings include associations between MMVD severity and polymorphisms in genes such as *NEBL*, *ACE*, *CDK6*, *HEPACAM2*, *COL5A1*, and *FAH*, as well as evidence implicating dysregulated TGF-β signaling, serotonin signaling, and extracellular matrix remodeling pathways. The current state of knowledge on the role of miRNA in the pathogenesis of MMVD was also summarized. Despite these findings, no specific high-penetrating mutation has been identified. MMVD is a complex, polygenic condition shaped by regulatory variants and breed-specific genetic bottlenecks. Comparative studies underscore the translational relevance of canine MMVD to human mitral valve disease, while genomic insights may be basis for the future selective breeding strategies and therapeutic approaches. Further large-scale, integrative studies combining genomics, transcriptomics, and functional validation are needed to clarify disease mechanisms and support targeted treatment in CKCS as well as the development of new breeding strategies and programs.

## Simple Summary

Myxomatous mitral valve disease (MMVD) is the most common heart condition in small breed dogs with Cavalier King Charles Spaniels being particularly predisposed. Nearly all Cavaliers develop this condition by the age of ten, and in many cases the disease begins much earlier. The disease affects the mitral valve and other valves, leading to progressive valve leakage and eventually heart failure. This review explains how genetic factors contribute to the development of myxomatous mitral valve disease in this breed. Several studies have identified candidate gene loci and regions, including *NEBL*, *ACE*, *CDK6*, *HEPACAM2*, *COL5A1*, and *FAH*, as well as signaling pathways such as TGF-β or 5-HT, that may influence the disease course. The role of miRNA in the pathogenesis of MMVD was also discussed. The genetic basis of the disease is complex and no single gene responsible for early onset of the myxomatous mitral valve disease has been found. Instead, a combination of hereditary risk factors, altered molecular pathways, and selective breeding practices appear to drive the high prevalence of MMVD in Cavaliers. Understanding these mechanisms will help veterinarians, breeders, and researchers to better diagnose, manage, and possibly prevent MMVD, ultimately improving the health and welfare of dogs.

## 1. Introduction

Myxomatous mitral valve disease (MMVD) is the most common heart disease in small and miniature breeds of dogs, to which the Cavalier King Charles Spaniel breed is particularly predisposed [1,2]. It accounts for 75% of all heart disease cases in dogs. MMVD affects more than half of the small breed dog population over 8 years of age, and even more in some breeds, such as CKCS [1]. According to the available literature, the incidence of MMVD in the CKCS breed reaches up to 100% above the age of 10 years [3,4,5]. The statistics describing the occurrence of the disease vary between different studies, but each of the available studies describes an exceptionally high prevalence of MMVD in the breed [6]. Data on the prevalence of the disease in the breed also vary geographically, which may be related to the different genetic pools of populations from different countries and the breeding programs conducted in them to reduce the incidence of MMVD. The disease can also occur in large and giant breed dogs and develop alone or in parallel with dilated cardiomyopathy [7,8]. The pathogenesis of this disease is complex and includes remodeling of the mitral annulus, valve leaflets, chordae tendineae, and ventricular and atrial myocardium [9]. The disease may involve only the mitral valve or also involve other valves in the heart [2]. The disease affects middle-aged and elderly dogs, but in some particularly predisposed breeds such as CKCS it can develop even at 1 year of age [4]. While environmental factors such as diet may influence the course of the disease, the influence of environmental conditions on predisposition or earlier development of MMVD has not yet been described either in CKCS or in other breeds of dogs [10,11]. In breeds not predisposed to heart disease, the influence of birth month on the risk of heart disease has also been described, although this has not been confirmed in breeds with a clear predisposition to heart disease such as CKCS [12]. Although MMVD occurs in both humans and animals, and the pathomechanisms responsible for the development of the disease are similar, their genetic backgrounds differ significantly, and genomic studies have not yet detected common genes responsible for the early development of MMVD [13,14].

## 2. Materials and Methods

In preparing this work, the focus was on articles from the last 15 years,; however, in the case of some important publications for the topic, older ones were also cited. The literature review was conducted based on the PubMed, Google Scholar, and Web of Science databases. The literature review was based on the keywords MMVD, CKCS, gene, polymorphism, mutation, SNP, and ROH in various combinations using operators “and” and “or”. Additionally, during the evaluation of individual articles, the bibliography of all works was assessed, thus supplementing the literature review used in our work. In this article, only works from peer-reviewed journals were cited; unpublished data and conference materials were excluded from the literature review.

## 3. Phenotype and Classification of MMVD

The diagnosis of the disease and its phenotyping are based primarily on echocardiography. Complementary tests include chest radiography, resting ECG, Holter ECG, and transesophageal echocardiography. Biomarkers such as NT pro BNP or TnI are also used in disease diagnostics. The MMVD phenotype includes the presence of a heart murmur, degenerative changes in the valve leaflets, insufficiency of the mitral valve and other heart valves, left-sided volume overload of the heart manifested by left ventricular and left atrial dilatation, and in advanced cases, pulmonary edema due to congestive heart failure (CHF). Among the echocardiographic parameters used to assess the degree of MMVD advancement, the LA:Ao ratio, normalized dimensions of the left ventricle and left atrium, or normalized left ventricular volume measured by the Simpson disk method are used. The mitral valve regurgitation gradient and mitral inflow profile are also of significant importance as predictors and indicators of congestive heart failure. These parameters reflect the pressure conditions prevailing in the heart chambers, allowing for the assessment of the degree of left ventricular volume overload. A common complication of the disease is the presence of ventricular and supraventricular extrasystoles, as well as the development of atrial fibrillation, which are diagnosed using ECG or Holter ECG. Increased concentrations of the above-mentioned biomarkers are also observed [1,2,3,4,5,15,16].

The main pathological changes in MMVD involve the valve leaflets and chordae tendineae with progressive structural degeneration. Those changes arise from a multifactorial process driven by chronic activation of valvular interstitial cells (VICs), dysregulated extracellular matrix (ECM) turnover, and altered mechanotransduction. Central to these events is upregulated transforming growth factor-β (TGF-β) signaling, which promotes a myxomatous rather than fibrotic remodeling pattern through VIC activation and matrix disorganization. Additional contributory pathways include serotonergic signaling, activation of the renin–angiotensin–aldosterone system (RAAS), oxidative stress, and inflammatory responses, all of which amplify ECM degradation, matrix metalloproteinase (MMP) and tissue inhibitor of metalloproteinases (TIMP) imbalance, and progressive leaflet thickening. These interacting molecular mechanisms collectively underpin the characteristic structural and functional deterioration of the mitral valve in MMVD. Histological changes in the mitral valve apparatus are usually described according to the Whitney classification, which grades the severity of myxomatous alterations based on the extent of extracellular matrix remodeling. Within the valve leaflets, the main changes include myxomatous expansion of the spongiosa and accumulation of glycosaminoglycans and proteoglycans causing nodular bulges. The fibrous layer becomes disorganized and thinned, with a concomitant loss of the lamellar architecture; elastic fibers show fragmentation and decreased density. Chordae tendineae undergo similar degenerative changes, including myxomatous infiltration, collagen disorganization, and progressive elongation [9,10,13,17,18,19]. Despite the different onset and rate of progression of MMVD in CKCS, the cellular and molecular mechanisms underlying the disease are the same across breeds, with CKCS exhibiting a similar pattern of myxomatous remodeling but manifesting it at an earlier age and with higher severity. Thus, the breed’s unique epidemiological and genetic features relate to disease severity and timing rather than to distinct pathophysiological processes [19].

There are several MMVD classification systems. Currently the most commonly used is the one developed by the American College of Veterinary Internal Medicine. Developed by a team of specialists, this is the most established classification system for the disease, based on various available diagnostic methods including echocardiography, thoracic radiography, clinical signs, and biomarkers. This classification divides the disease into four stages from A to D, where stage A includes patients with a clear breed predisposition, stage B includes asymptomatic patients with the presence of structural changes in the heart muscle, and stages C and D collect patients with signs of congestive heart failure. Stage C patients are dogs that respond positively to standard doses of medications (pimobendan, furosemide, spironolactone, ACE-I, etc.) for the treatment of congestive heart failure, while stage D collects patients refractory to standard doses of medications used to treat CHF. It precisely describes the management at each stage of the disease, providing detailed recommendations for diagnosis, monitoring, pharmacological therapy, and interventional options at each stage. To classify dogs into specific stages of the disease, clinical symptoms such as cough or exercise intolerance are assessed, the presence and intensity of heart murmurs are checked by auscultation, the vertebral heart score is assessed in radiological examinations, the size of the heart chambers and the pressure conditions are assessed by echocardiography, and the concentration of serum NT-proBNP is also assessed [1,2,20,21,22].

The relatively recently developed MINE score based only on echocardiographic findings is also gaining popularity, with the advantage of including prognosis based on retrospective patient survival times. The MINE score is primarily used in patients with ACVIM stage B2, allowing for the assessment of prognosis and predicted survival time based on a combination of three basic echocardiographic parameters: the LA/Ao ratio, LVIDDn, and mitral inflow E-wave velocity. This classification therefore serves more as a supplement to the commonly used ACVIM classification rather than a standalone tool. Its main advantage is the numerical representation of the severity of changes, allowing for better prediction of an impending heart failure episode [23].

## 4. Breed Predispositions and Inheritance

Breeds that are particularly predisposed to early development of MMVD include the CKCSs, Chihuahuas, Cocker Spaniels, Whippets, and Dachshunds [24]. The available literature also describes a sex-linked predisposition, with males having an earlier, more frequent, and more severe course of a disease. A significant hereditary factor for MMVD has also been demonstrated. This was described, among others, by Lewis et al. in a population of 1252 CKCS dogs. Based on auscultation and the presence of murmur at a young age, the high heredity of the disease was confirmed, demonstrating its genetic basis [25]. The low heterozygosity rate in this breed described by Mellanby et al. also contributes to the increased heritability of MMVD and other diseases [26]. Breeding techniques involving inbreeding practices to maintain the breed standard lead to increased levels of homozygosity and therefore an increased risk of disease.

Due to such a high breed predisposition, some breeding associations have introduced breed-specific breeding programs that include screening tests aimed at eliminating from breeding individuals with a greater predisposition to the early development of MMVD. The results of programs conducted in Denmark and Sweden have been published with different results (Table 1). The breeding program implemented in Sweden based solely on annual cardiac auscultation did not produce statistically significant results. In this program, individuals over four years of age were permitted to breed, provided they had not developed a heart murmur confirmed by auscultation within the previous eight months. Additionally, individuals from two years of age were permitted if their parents also had no heart murmur. Individuals whose parents had developed a heart murmur before the age of four were excluded from breeding. No further breeding restrictions were imposed on males without a heart murmur after the age of seven. Despite the relatively restrictive requirements for qualifying dogs for breeding, this study did not confirm the effectiveness of the breeding program in Sweden, while confirming the high prevalence of the disease in the breed [27]. The Danish Kennel Club has implemented a much more restrictive breeding program based on annual echocardiography and cardiac auscultation. Only dogs free of the disease were allowed for breeding; dogs needed to be re-examined at the ages of 18 months, 4 years, and 6 years. In the Danish program, in addition to auscultation, echocardiography assessed the presence and degree of mitral regurgitation, as well as mitral valve prolapse. Only dogs meeting the established criteria were approved for breeding, and the test results were made publicly available to breeders. Over a period of 8–10 years, this program has statistically significantly reduced the prevalence of MMVD in CKCS, and the age of onset of the disease was delayed by up to 2 years. In 2010–2011, the dogs examined with the Danish Kennel Club breeding protocol had a 73% lower risk of heart murmur compared with dogs examined in 2002–2003 [28].

Another disease to which CKCS dogs are particularly predisposed is Chiari malformation. A study conducted by Ancot et al. in 2018 identified two candidate loci on CFA22 and CFA26 that may be responsible for the development of Chiari malformation in CKCSs [29]. A further study in 2024 by Bach et al. investigated the potential association between the development of Chiari malformation and the presence of MMVD. However, no association was found between the development of MMVD and the presence of Chiari malformation [30]. The lack of association between the occurrence of MMVD and Chiari malformation indicates different genetic mechanisms determining the occurrence of these diseases.

The high incidence of MMVD in certain breeds, especially in CKCSs, which show an exceptionally early and severe course of MMVD, has led to the need to investigate the genetic basis of early development of MMVD in order to conduct more targeted breeding efforts to reduce its incidence. Research conducted to date confirms the polygenic nature of the disease [31]. The polygenic nature of the disease poses a particular diagnostic and breeding challenge; therefore, studies were carried out to evaluate genes encoding proteins involved in metabolic pathways involved in the pathogenesis of MMVD.

## 5. Physiological and Molecular Markers Associated with MMVD

### 5.1. RAA System

The renin–angiotensin–aldosterone system (RAAS) is a crucial central neurohormonal driver of disease progression in cases of myxomatous mitral valve disease. Chronic left-sided volume overload caused by mitral valve regurgitation lowers the effective arterial perfusion pressure and activates baroreceptor pathways, stimulating renin release and further generation of angiotensin II and aldosterone. In addition to their vasoconstrictor effects and renal sodium and water retention, angiotensin II and aldosterone exert direct effects on the myocardium and valves, promoting fibroblast activation, extracellular matrix remodeling, and electrical/structural remodeling of the heart, which collectively exacerbate regurgitation and predispose to further heart insufficiency and heart failure. These maladaptive responses occur in both the circulating and tissue (cardiac) RAAS and provide a strong mechanistic reason for RAAS-modulating therapies in dogs with MMVD [32,33].

A series of studies conducted mainly by Meurs et al. have identified an intronic *ACE* gene variant (9:11507816:G>A) which was considered to be associated with lower circulating ACE activity in several breeds, including CKCSs [32,33,34,35]. The studied populations of CKCS dogs showed a high prevalence of this variant. In a population of 73 CKCS dogs, this variant was detected in 48 dogs, of which 43 were homozygous and 5 were heterozygous. The remainder of the studied population were wild-type homozygotes [32]. In another study involving dogs of various breeds, 13 CKCS dogs were examined, of which 7 were heterozygous and 4 were homozygous, with only 2 being wild-type [35]. Initially, these studies considered the potential impact of the presence of polymorphism on the therapeutic effect of ACE inhibitor treatment in the course of MMVD. Importantly, dogs carrying this variant still demonstrate pharmacologic ACE suppression after ACE inhibitor administration, and, to date, a consistent, clinically meaningful difference in therapeutic response to ACE inhibition between variant-positive and wild-type dogs has not been proven [36]. Thus, genotype alone should not be used to predict ACE-inhibitor efficacy in individual patients. Nonetheless, multiple lines of evidence show that aldosterone breakthrough (re-emergence or persistence of aldosterone production despite ACE inhibition) occurs in a substantial subset of dogs with MMVD, reflecting activation of non-ACE pathways and tissue RAAS [32,36,37,38]. Prospective RAAS profiling work and clinical studies therefore support sequential blockade of the RAAS by combining an ACE inhibitor with a mineralocorticoid receptor antagonist such as spironolactone, particularly once congestion is present. This approach is consistent with the current ACVIM consensus statement, which endorses spironolactone as part of the standard regimen for dogs with MMVD in stage C and with congestive heart failure [2].

### 5.2. Nebulette Protein

Nebulette protein, encoded by the *NEBL* gene, is a cardiac, nebulin-family actin-binding protein that anchors thin filaments to the Z-disc and links the contractile apparatus to costameres, thereby supporting sarcomere architecture and force transmission. In cardiomyocytes it localizes predominantly to Z-discs of atrial and ventricular myofibrils and participates in mechanotransduction; perturbing nebulette disrupts myofibril assembly, desmin organization, and Z-disc integrity. Studies in humans and experimental models have previously shown an association of *NEBL* gene mutations with the development of cardiomyopathy and endocardial fibroelastosis. However, in humans, an association of *NEBL* gene mutations with MMVD has not yet been described [39,40,41]. In dogs, the *NEBL* gene is located on chromosome 2 (CFA2) [39].

In CKCS and other breeds of dogs, a whole-genome study by Axelsson et al. nominated a set of intronic variants at the *NEBL* locus with regulatory potential in heart-derived cells and showed that two candidates were associated with reduced nebulette isoform expression in papillary muscle, leading the authors to propose that compromised papillary muscle integrity could predispose to mitral regurgitation and MMVD [42].

Building on this, Mead et al. genotyped 180 Australian CKCSs at the a priori risk loci (NEBL1–3 on CFA2) and demonstrated associations between *NEBL* variants and echocardiographic severity indices (e.g., LA:Ao ratio, weight-normalized LVIDd), supporting a role for NEBL haplotypes in earlier and more severe course of MMVD in this breed [43]. While these data strengthen the biological plausibility of *NEBL* as a modifier locus, they point to regulatory, non-coding mechanisms rather than a single high-penetrance coding mutation; thus, validation in larger cohorts and functional assays in canine valve/papillary muscle tissue remain priorities before clinical or breeding decisions rely on *NEBL* genotyping. Contrary to humans, no association of *NEBL* gene mutations with heart diseases other than MMVD in dogs, such as cardiomyopathies, has been described so far.

### 5.3. TGF-β

Myxomatous mitral valve disease (MMVD) in dogs and humans features leaflet thickening and distortion, disorganized extracellular matrix (ECM), and a phenotypic switch of quiescent valve interstitial cells (qVICs) toward an activated, myofibroblast-like state (aVICs) [44,45,46]. Across disease grades, transcriptomic profiling shows that dysregulated TGF-β signaling is a dominant driver of these processes, consistent with upregulated ligand expression and downstream pathway activation within MMVD-affected valves [46,47,48].

Mechanistically, TGF-β promotes ECM remodeling through canonical SMAD2/3 signaling and via non-canonical cascades (e.g., ERK, p38, and PI3K/AKT/mTOR), thereby increasing matrix synthesis, altering collagen/elastin architecture, and sustaining the aVIC phenotype. Recent studies have specifically implicated PI3K/AKT/mTOR as a TGF-β-responsive axis in MMVD valves, underscoring the breadth of TGF-β pathway activation beyond SMADs [47,49].

In vitro studies with canine mitral VICs demonstrate that TGF-β stimulation induces α-SMA expression and a profibrotic effect; crosstalk with serotonin (5-HT) signaling can further amplify this activation, highlighting convergent profibrotic inputs relevant to disease progression [45,49].

At the tissue level, severe canine MMVD is associated with altered expression of TGF-β, matrix metalloproteinases (MMPs), and their tissue inhibitors (TIMPs) within mitral leaflets and myocardium, with parallel changes detectable in plasma; several of these molecular changes correlate with echocardiographic indices of disease severity. These findings support a model in which TGF-β-driven imbalance between MMPs and TIMPs contributes to ECM disorganization and leaflet deformity [44,50].

Finally, comparative pathology and focused reviews converge on a shared, central role for TGF-β signaling in canine MMVD and human mitral valve prolapse, reinforcing the translational value of canine disease for interrogating TGF-β-targeted strategies [46,47,48].

An important conceptual aspect of TGF-β biology in MMVD is that its downstream effects are highly context-dependent and shaped by the unique cellular and extracellular environment of the mitral valve. In many tissues, TGF-β activation promotes classical fibrosis characterized by dense collagen deposition and progressive tissue stiffening. In contrast, in myxomatous mitral valve degeneration the same canonical pathway results in a distinct fibromyxoid phenotype, marked by proteoglycan accumulation within the spongiosa, disruption of collagen–elastin architecture, and a transition of quiescent VICs into proliferative, ECM-producing aVICs [44,45,46]. Transcriptomic and immunohistochemical studies have shown that this divergence reflects the combined influence of altered mechanotransduction, the inherent matrix composition of valvular tissue, and parallel activation of non-canonical cascades such as ERK and PI3K/AKT/mTOR, which amplify TGF-β-driven remodeling [47,49]. Crosstalk between TGF-β and serotonin (5-HT) signaling further reinforces this fibromyxoid pattern, biasing leaflet remodeling away from classical fibrosis and toward the characteristic myxomatous changes observed in both canine and human MMVD [45,50]. Recognizing this context-dependent behavior of TGF-β is essential for explaining why MMVD progresses through myxomatous leaflet thickening and deformation rather than the formation of a fibrotic scar, despite the involvement of canonical TGF-β signaling pathways.

### 5.4. HEPACAM2, CDK6, FAH

A study published by Madsen et al. identified another two candidate regions on chromosomes 13 and 14 (CFA13, CFA14) as potentially responsible for the early development of MMVD in CKCS [51]. Subsequent studies have not yet identified the specific variants responsible for disease development. Related regions were later investigated by Bionda et al.

In a case–control genomic study of 33 CKCSs (16 early-onset cases < 5 years; 17 older/mild controls), Bionda et al. applied complementary selection signature analyses-Wright’s fixation index (F_ST)-cross-population extended haplotype homozygosity (XP-EHH), and runs of homozygosity (ROH) on 230K SNP array data. They mapped the top-1% signals to ten “consensus genes” distributed across CFA3, CFA11, CFA14, and CFA19; among these, *HEPACAM2* and *CDK6* (both on CFA14) and *FAH* (CFA3) emerged as the most compelling because of additional ROH support. Notably, ROH encompassing *HEPACAM2* and *CDK6* occurred in ~50% of early-onset cases versus ≤9% of controls, while ROH including *FAH* were present in ~80% of early-onset cases versus ~40% of controls—patterns consistent with selection or enriched risk haplotypes in more severe/early disease. Pathway enrichment placed several consensus genes (including *CDK6*) within TGF-β-related networks, aligning with established profibrotic signaling in MMVD. However, the study did not identify specific causal coding variants; rather, it prioritized loci for follow-up in larger, longitudinal cohorts [47,48,52].

From a mechanistic standpoint, these candidates are biologically plausible but remain hypothesis-generating. *CDK6* encodes a G1/S cell-cycle kinase that regulates pRB phosphorylation and proliferation; altered control of VIC proliferation and phenotype is a hallmark of degenerative valve disease. It also interacts with *CDKN2B* (cyclin-dependent kinase inhibitor 2B), whose expression is induced by TGF-β and is associated with coronary heart disease in humans. *HEPACAM2* encodes an immunoglobulin superfamily adhesion protein with reported roles in mitosis and cell-cell adhesion, processes relevant to leaflet tissue integrity; its dysregulation has been observed in canine tissues, supporting a potential role in valvular remodeling. *HEPACAM2* also interacts with *FGFR1* (fibroblast growth factor receptor 1). *FAH* encodes fumarylacetoacetate hydrolase, the terminal enzyme in tyrosine catabolism; although classically linked to hepatic disease, perturbations in amino acid metabolism and downstream oxidative stress could, in principle, influence valvular cell homeostasis. *FAH* also interacts with *ADAMTSL4* (ADAMTS like 4). *HEPACAM2*, *CDK6*, and *FAH* delineate promising genomic neighborhoods associated with early or severe MMVD in CKCS via selection signatures and ROH, with functional annotations pointing toward cell-cycle/adhesion pathways intersecting TGF-β signaling [53]. Replication in larger, multi-center cohorts with uniform phenotyping, fine-mapping of causal variants, and in vitro/ex vivo validation in canine VICs and leaflet tissue are the necessary next steps before any clinical or breeding applications are considered.

### 5.5. Collagen

In both humans and dogs, disturbances in collagen metabolism and extracellular matrix remodeling represent key pathological features of myxomatous mitral valve disease. In a 2017 study by Meurs et al., using a population of 10 dachshunds and 10 CKCS dogs, genes involved in the pathogenesis of MMVD in humans were evaluated for the presence of variants potentially predisposing to MMVD. Among other things, genes for various collagen types were assessed. In the studied CKCS population, a nonsense variant at location 9:50759457 was detected in the collagen type V alpha 1 (*COL5A1*) gene in nine dogs. Its impact was described as benign; however, the exact impact of the variant was not described. Two other intronic variants were also detected in this study, but they did not meet the criteria for conservation or were predicted to change or create a splice site [14].

In the study by Torres-García et al., a population of 50 Poodle dogs diagnosed with MMVD versus 80 controls was evaluated for two intronic variants within the *COL1A2* gene (rs9006567 and rs22372411) [54]. The *COL1A2* c.1351-46C > T variant (rs22372411) was described in this study as predisposing to MMVD, which is consistent with the candidate region previously described by Madsen et al. in the Cavalier King Charles Spaniel population [51,54].

No data on other potential loci within the collagen genes have been published to date for Cavalier King Charles Spaniels.

### 5.6. Serotonin Transporter (5-HT)

Serotonin (5-HT) signaling is also a key modulator in canine MMVD, linked to the TGF-β pathway. In a candidate gene study in Maltese dogs, Lee et al. sequenced all 5-HT transporter (*SERT*) gene exons in animals with degenerative mitral valve disease and healthy controls, identifying six polymorphisms, including three nonsynonymous coding variants, that were predicted by in silico modeling to impair protein function. These variants were detected more frequently in dogs with MMVD compared to controls, leading the authors to hypothesize that impaired *SERT* function, and consequently altered 5-HT transport at the valve surface, may contribute to susceptibility or earlier onset of MMVD in Maltese dogs [55]. Reimann et al. studied a CKCS population in different stages of MMVD and genotyped in detail three *SERT* polymorphisms previously associated with MMVD in Maltese dogs. None of these polymorphisms was detected in any CKCS dog, and no alternative *SERT* variants with clear pathogenic significance were identified. Furthermore, in the CKCS population studied, serum 5-HT concentrations were primarily associated with platelet counts, rather than with MMVD stage or treatment, suggesting that Maltese *SERT* risk variants are not common to CKCS and are unlikely to explain the high burden of MMVD in this breed. These results support a model in which serotonergic pathways (and their interaction with TGF-β signaling) contribute significantly to myxomatous valve remodeling, but the underlying genetic architecture of 5-HT transport appears to be strongly breed-dependent [56]. Furthermore, when comparing other loci from the genomic study in Maltese dogs with the studies included in this literature review, despite the description of variant changes in similar metabolic pathways, no such common variants were detected in CKCS dogs as potential candidate variants for MMVD [57].

### 5.7. miRNA

MicroRNAs (miRNAs) constitute a key layer of post-transcriptional regulation in mitral valve interstitial cells (VICs), orchestrating multiple signaling networks that drive the initiation and progression of MMVD. In valvular heart diseases, miRNAs have been recognized as central modulators of extracellular matrix (ECM) dynamics, fibrosis, inflammation, and cell differentiation [58,59]. Transcriptomic and miRNA-sequencing studies performed in dogs-including CKCS-have consistently demonstrated altered expression of numerous miRNAs in advanced stages of MMVD, particularly those associated with TGF-β-dependent profibrotic signaling, ECM remodeling, and the activation of the myofibroblast (aVIC) phenotype [60,61,62,63,64,65,66]. Among these, members of the miR-29 family-canonical inhibitors of collagen and fibrillin synthesis-are notably dysregulated in degenerative canine valves, and their reduced activity contributes directly to ECM expansion characteristic of myxomatous lesions [58,62]. Elevated expression of miR-21, a potent amplifier of TGF-β/SMAD signaling, has been repeatedly demonstrated in canine MMVD and correlates with disease severity and the persistence of aVIC-mediated fibromyxoid changes [58,59,62]. Additional miRNAs implicated in MMVD include miR-133, involved in cytoskeletal organization and mechanotransduction, and miR-30, which normally suppresses fibrogenic pathways; both exhibit altered activation patterns that favor pro-remodeling processes during disease progression [62].

Beyond valve-specific expression profiles, several studies have assessed circulating miRNAs as minimally invasive diagnostic and prognostic biomarkers. Altered levels of multiple miRNAs, including miR-17, miR-20a, miR-30d, and let-7c, have been detected in dogs with naturally occurring MMVD and congestive heart failure (CHF), supporting their role as systemic indicators of cardiac remodeling [61,63,64,65]. Recent work suggests that specific miRNA signatures may predict early MMVD development or stratify disease severity, highlighting their potential translational relevance for clinical screening—particularly in genetically predisposed breeds such as CKCS [60,66].

Despite extensive evidence for pathogenic shifts in miRNA expression, no mutations, SNPs, or structural variants in miRNA-coding regions or their canonical regulatory elements have been identified in CKCS that would predispose directly to MMVD. Current findings indicate that miRNA dysfunction in this breed is secondary, emerging from a molecular milieu dominated by chronic TGF-β activation, altered mechanotransduction, oxidative stress, and VIC phenotypic plasticity, rather than resulting from primary genetic defects in miRNA loci. Integrative analysis of RNA-seq, miRNA-seq, and epigenetics may reveal hidden non-coding regulatory pathways essential for understanding the predisposition of this breed to MMVD [45,58,59,60,61,62,63,64,65,66].

## 6. Conclusions

Myxomatous mitral valve disease (MMVD) remains a major health problem in Cavalier King Charles Spaniels due to its early onset, progressive course, and strong hereditary component. Recent advances in molecular genetics have identified several genes and regulatory pathways potentially involved in the pathogenesis and phenotypic expression of the disease, including *NEBL*, *HEPACAM2*, *CDK6*, *FAH*, *COL5A1*, *SERT*/5-HT, and *ACE*. In addition, the central role of TGF-β signaling and extracellular matrix remodeling proteins such as MMPs and TIMPs underscores the multifactorial nature of MMVD (Table 2). Although certain polymorphisms have been associated with disease severity, further studies are needed to confirm their predictive value and utility in selective breeding programs. miRNAs also play a key role in the pathogenesis of MMVD. While still poorly understood, they have high diagnostic potential as biomarkers. It is also crucial to determine whether there are mutations responsible for differences in the role of miRNAs in MMVD pathogenesis compared to other breeds. A comprehensive understanding of genetic factors may contribute to the development of targeted therapies and reduce the incidence of the disease through genomic studies and responsible breeding strategies.

The available literature mentioned in this review strongly supports the view that MMVD is a complex, multigenic disease, with no single high-penetrance mutation responsible for early onset and severe course of the disease identified to date. Instead, a combination of regulatory variants, altered signaling pathways, and breed-specific genetic bottlenecks contribute to disease expression. The polygenic nature of the disease poses a particular breeding challenge. Even with the knowledge currently available, it remains extremely difficult to identify individuals highly predisposed to the disease and eliminate them from breeding. To date, the most effective breeding programs rely on clinical examination of the patient and diagnostic imaging, rather than on the animals’ genetic profile. This indicates that integrative genomic approaches, combining genome-wide studies, transcriptomics, and functional validation in cardiac tissue, will be essential for identifying and clarifying mechanisms of early onset of the disease in CKCS.

## Figures and Tables

**Table 1:** Comparison of the Swedish and Danish breeding programs for reducing MMVD prevalence in Cavalier King Charles Spaniels [27,28].

	Swedish Breeding Program	Danish Breeding Program
Method of screening	Annual cardiac auscultation only	Auscultation + echocardiography
Minimal age for breeding	Dogs ≥ 4 years may be bred if free of murmur within the last 8 months; dogs ≥ 2 years allowed if both parents were murmur-free at ≥4 years	Dogs must be free of cardiac disease at each required examination; no breeding permitted before completing scheduled checks
Additional parental restrictions	Dogs whose parents developed a murmur before 4 years were excluded from breeding	Results of all screened dogs were publicly available, enabling selection against affected family lines
Follow-up examinations	Mandatory within 8 months before mating; males over 7 years of age without murmur may be bred without further evaluation	Required at 18 months, 4 years, and 6 years to maintain breeding eligibility
Overall effectiveness	Considered ineffective in reducing MMVD prevalence	Considered effective, demonstrating clear epidemiological improvement

**Table 2:** Summary of the candidate loci described so far to be predisposing to early-onset and severe course of MMVD in CKCS.

Locus/Gene	Location	Proposed Effect/Mechanism	References
*ACE*	CFA9	- Lower circulating ACE activity - No effect on ACE inhibition with ACE-I - Aldosterone breakthrough despite ACE-I suggests non-ACE/tissue RAAS leading to ongoing fibroblast activation, ECM remodeling and electrical, and structural remodeling of the valve and myocardium -Variant present in various breeds	[29,31,32,33,34]
*NEBL1-3* (nebulette)	CFA2	- Intronic/regulatory variants at *NEBL* are linked to reduced nebulette isoform expression in papillary muscle - Impaired sarcomere and mechanotransduction and papillary muscle dysfunction - Association with higher LA:Ao ratio and LVIDd supports a modifier effect on severity/earlier onset of MMVD - Variant described in CKCS and Dachshunds; commercial test available for Cavaliers	[39,40]
*HEPACAM2*	CFA14	- ROH enrichment at/near *HEPACAM2* suggests selection/enriched risk haplotypes - Hypothesized adhesion/mitotic dysregulation in VICs or leaflet cells leading to aberrant tissue integrity and maladaptive remodeling under hemodynamic stress	[48,49]
*CDK6*	CFA14	- ROH signal and pathway placement within TGF-β - Dysregulated VIC proliferation/activation - Sustaining the myofibroblastic (aVIC) phenotype and ECM remodeling that thickens/deforms leaflets	[48,49]
*FAH*	CFA3	- ROH enrichment - Interacts with *ADAMTSL4* - Mechanistically, altered tyrosine catabolism could raise oxidative/alkylating stress, disturbing VIC homeostasis and matrix turnover, thereby amplifying leaflet degeneration	[49]
*COL1A2/COL5A1* (collagen genes)	CFA14/CFA9	- *COL5A1* intronic and nonsense variants reported in CKCS - *COL1A2* variant associated with MMVD in Poodles; limited data in CKCS - Collagen dysregulation contributes to ECM architecture changes	[14,51,54]
SERT	CFA9	- Altered 5-HT transport may contribute to susceptibility or earlier onset of MMVD - Maltese dogs show putative risk variants; none replicated in CKCS - Serotonin—TGF-β interaction remains important mechanistically despite lack of breed-specific variants	[55,56,57]
miRNA	Non-coding genome	- Dysregulation of miRNAs (e.g., miR-21, miR-29, miR-133, miR-30, let-7 family) promotes TGF-β-driven activation of VICs and ECM remodeling - No pathogenic miRNA-coding mutations identified in CKCS - Likely secondary to TGF-β activation and biomechanical stress	[58,59,60,61,62,63,64,65,66]
TGF-β signaling network (pathway)	Multiple loci	- MMVD leaflets show upregulated TGF-β signaling, α-SMA induction in VICs, and MMP/TIMP imbalance, driving ECM disorganization, leaflet thickening, and deformity - Acts as a genetic modifier pathway that may intersect with CDK6 and other candidates - No single causal SNP established	[44,45,47]

## Data Availability

No new data were created or analyzed in this study. Data sharing is not applicable to this article.
